# Opportunities and Challenges for Digital Social Prescribing in Mental Health: Questionnaire Study

**DOI:** 10.2196/17438

**Published:** 2021-03-09

**Authors:** Shivani Patel, Gerry Craigen, Mariana Pinto da Costa, Becky Inkster

**Affiliations:** 1 South London and Maudsley NHS Trust London United Kingdom; 2 Department of Psychiatry, Faculty of Medicine University of Toronto Toronto, ON Canada; 3 Centre for Mental Health University Health Network – Toronto General Toronto, ON Canada; 4 Institute of Psychiatry, Psychology and Neuroscience King’s College London London United Kingdom; 5 Institute of Biomedical Sciences Abel Salazar University of Porto Porto Portugal; 6 Department of Psychiatry University of Cambridge Cambridge United Kingdom; 7 Finance and Economics Programme The Alan Turing Institute London United Kingdom

**Keywords:** mental health, technology, psychiatry, mobile phone

## Abstract

**Background:**

The concept of digital social prescription usually refers to social prescriptions that are facilitated by using technology. Tools that enable such digital social prescriptions may be beneficial in recommending nonmedical activities to people with mental illness. As these tools are still somewhat novel and emerging, little is known about their potential advantages and disadvantages.

**Objective:**

The objective of this study is to identify the potential opportunities and challenges that may arise from digital social prescriptions.

**Methods:**

We developed a qualitative questionnaire that was disseminated through social media (Facebook and Twitter). A purposive sample targeting *digital mental health experts* and nonexperts was approached. The questionnaire asked participants’ views about digital social prescription; the core elements linked with a definition of digital social prescription; and the strengths, weaknesses, opportunities, and threats associated with digital social prescription.

**Results:**

Four core elements were recommended to define the concept of digital social prescription: digital, facilitate, user, and social. The main strength identified was the possibility to rapidly start using digital social prescription tools, which were perceived as cost-effective. The main weaknesses were their poor adherence and difficulties with using such tools. The main opportunities were an increased access to social prescription services and the prevention of serious mental illness. The main threats were certain groups being disadvantaged, patients being subject to unintended negative consequences, and issues relating to confidentiality and data protection.

**Conclusions:**

Although digital social prescriptions may be able to effectively augment the social prescriptions, a careful consideration of practical challenges and data ethics is imperative in the design and implementation of such technologies.

## Introduction

### Background

The idea of health care professionals prescribing activities to their patients has been around since the 1990s when contemporary exercise referral schemes were first created [1]. The term *social prescription* has since been defined as “a means of enabling general practitioners (GPs) and other frontline health care professionals to refer people to ‘services’ in the community instead of offering only medical solutions” [2]. Social prescribing services are typically offered by voluntary and community sector organizations and usually involve a person who supports people to access local activities [3]. Examples of activities may range from traditional formalized programs such as smoking cessation programs to exercise, cooking classes, and befriending services [4-6]. The benefits of social prescribing have previously been explored, with studies suggesting a reduction in GP consultations and accident and emergency department attendance when social prescribing services are working well [7]^.^ and a reduced requirement for psychiatrists and mental health nurse consultations [8]. The term *digital social prescription* has previously been described as “any digital solution, technology, information or electronic system that enables social prescribing” [9].

Digital technologies have become increasingly pervasive within the society [10], and our dependence on interactive technologies for the delivery of health care has been particularly important during the global COVID-19 pandemic [11]. Interactive technologies have successfully enabled changes in human attitudes and behaviors [12,13], and the use of this technology for social prescription could offer a health benefit to our modern society. Currently, digital social prescription tools (DSPTs) used in the United Kingdom are used for patients with physical health comorbidities. DSPTs, such as those developed by Evergreen Life [14] and Elemental [15], use electronic patient records and community directory software to match nonmedical activities that have been shown to benefit a patient’s medical condition. The matching process involves using an algorithm designed to match activities to a patient based on their preferences, comorbidities, and locality. This process aims to tailor nonmedical interventions to the needs and preferences of the patient in a sophisticated and efficient manner.

### Objective

The objective of this study is to collect the views of both experts and the general public on digital social prescription while focusing on the core elements that should base the concept of digital social prescription and identify the potential benefits and challenges that may arise from digital social prescriptions.

## Methods

### Study Design

This study includes a qualitative questionnaire (Multimedia Appendix 1) with views of both experts and nonexperts on the potential use of digital social prescriptions.

### Instrument

The questionnaire started with a short introduction of digital social prescriptions, including a diagram on how it might work in practice. The questionnaire asked participants’ views of digital social prescription; the core elements linked with a definition of digital social prescription; and the strengths, weaknesses, opportunities, and threats (SWOT) associated with digital social prescription.

### Data Sampling and Collection

The instrument targeted digital mental health experts and nonexperts. Experts were selected from a purposive sample of researchers who had published in the *Journal of Medical Internet Research* on a topic relating to digital mental health in the last 5 years and were contacted by email. Nonexperts were approached through social media platforms (Facebook and Twitter).

### Data Analysis

We used content analysis [16] and the SWOT framework to analyze responses from participants. SWOT frameworks are commonly used in strategic analysis to analyze the internal (strengths and weaknesses) and external (opportunities and threats) factors relating to a project concept or idea [17]. The first author (SP) coded all the material, and the third author (MP) reviewed all the data to ensure the consistency and credibility of the coding and grouping [18].

## Results

### Sociodemographic Data of Participants

Our sample consisted of 22 *nonexpert* participants and 22 *expert* participants (Table 1).

**Table 1:** Demographics of the sample.

Demographics		Expert, n (%)	Nonexpert, n (%)
**Age (years)**			
	0-20	0 (0)	2 (9)
	20-30	12 (55)	14 (64)
	30-40	8 (36)	4 (18)
	40-50	2 (9)	1 (4.5)
	50+	0 (0)	1 (4.5)
**Gender**			
	Male	8 (36)	10 (45.5)
	Female	69 (59)	12 (54.5)
	Nonbinary	23 (5)	0 (0)
**Nationality**			
	British	6 (27)	19 (86.5)
	Canadian	4 (18)	1 (4.5)
	Indian	0 (0)	1 (4.5)
	Greek	0 (0)	1 (4.5)
	Dutch	2 (9)	0 (0)
	American	8 (41.5)	0 (0)
	Australian	1 (4.5)	0 (0)
**Occupation**			
	Mental health professional (doctor, psychologist, or mental health worker)	0 (0)	8 (36)
	Student	1 (4.5)	12 (54.5)
	Researcher	21 (95.5)	1 (4.5)
	Unemployed	0 (0)	1 (4.5)

### Definition of Digital Social Prescription

Expert and nonexpert participants were asked to provide a definition of digital social prescriptions. For both groups, the responses gathered identified four core elements: (1) digital, (2) facilitate, (3) user, and (4) social (Table 2).

As a result, the following definition is proposed: *digital social prescription refers to social prescriptions that have been facilitated through the use of technology, such as mobile phone apps or online platforms intended to benefit its users*.

We used the terms *digital social prescription tools* and *digital platforms* interchangeably to reflect the views of our participants.

The findings from our SWOT analysis are reported in Textbox 1 and Textbox 2.

**Table 2:** Expert participants’ (N=22) and nonexpert participants’ (N=22) responses to the question “How would you define digital social prescription?” grouped by core element.

Participant type	Words from participants’ responses			
	Digital	Facilitate	User	Social
Expert	Digital Technology Web-based platforms	Use Facilitate	Recommended as a part of health care Prescribed by a clinician Recommended to patients	Social prescription
Nonexpert	Technology App Digital platform	Facilitate Link Allow	Self Patients Doctors	Nonmedical activities Social prescription

**Strengths, weaknesses, opportunities, and threats analysis of responses from expert participants (n=22).**

**Strengths**

- Time (n=13)
  Quick to start
  Quick to download
- Easy to use (n=11)
  Intuitive for users
  Easy to use
- Social connection (n=7)
  Social connection in local area
  Social connection in area

**Weaknesses**

- Loss of interest (n=15)
  Fatigue
  High drop-off rate
  Lack of continuity
- Hard to use (n=8)
  Technical difficulties to use
  Not acceptable to disadvantaged groups—lower socioeconomic groups, older people, and people with physical health comorbidities
- Authenticity of participation (n=1)
  Interference from bots and trolls
- Difficulty in remaining updated (n=4)
  Difficult to keep up with new technologies
  Difficulties with maintaining lists

**Opportunities**

- Improved access (n=15)
  Access for more people
  Greater access if done equitably
  Access to care for poorer groups in the society
  Access for hard-to-reach groups—poor mobility and poor socioeconomic groups
- Loneliness (n=7)
  Help to combat isolation
  Target loneliness
- Resource efficiency (n=2)
  Help to free up resources that can be redirected toward significant mental illness

**Threats**

- Privacy and confidentiality relating to data (n=14)
  Privacy of data
  Use and storage of data
- Widening the health gap (n=5)
  Widen the gap between those who can afford technologies and those who cannot
  Digital divide exacerbating health inequalities
- Not accepted by establishment (n=1)
  Seen as a fad by traditional clinicians

**Strengths, weaknesses, opportunities, and threats analysis of responses from nonexpert participants (n=22).**

**Strengths**

- Cost (n=17)
  Cheap or cost-effective
  Requires fewer human resources to be involved in the process as it uses an algorithm for matching
- Time (n=10)
  Quick
  Reduce the waiting time between patient expressing an interest and being able to start an activity
- User experience (n=9)
  System more transparent for patients as they can track their social prescribing referral throughout the process
  “On-demand” service
  Younger generations might find it easier to engage
  All that there is to offer in one place
- Local (n=8)
  Able to easily identify activities close to patient’s location
  Digital social prescribing will match patient with local activities, allowing patients to feel more connected to their community
  Easy to find available activities (n=5)
  Update activities quickly
  Greater variety of activities and easier to keep a register
- Efficiency (n=3)
  Less paperwork

**Weaknesses**

- Difficulty in using the tool (n=19)
  Difficulties in using it
  Older generation and very ill patients might find it difficult to use such tools
  Language barriers
- Poor engagement (n=14)
  People not turning up to activities
  People not using it over longer periods
  Not everyone understands the intended goal
  May not be culturally appropriate
- Lack of human connection (n=13)
  Patients feel they are not being listened to
  Patients might be distrustful, lack of link worker to help with building trust
  No function for support workers to provide guidance
  Patient expectations for the management of problem (n=10)
  “Tech solution” might put people off
  People may feel this is not an appropriate response
  Mismatch between patients’ expectations of an activity and the reality
  People might be offended that they are asked to use a digital app instead of being able to talk to a health care professional in the first instance
- Delay in appropriate management (n=1)
  Delay in treatment
  Problems with maintaining lists of activities (n=3)
  Problems with social prescription—directories with activities are not free of errors or comprehensive
  Community centers are paper based
  Activities do not get listed
  Cost of keeping this system updated

**Opportunities**

- Greater access (n=20)
  Greater access to activities
  Allow for a more widespread uptake of social prescription
- Address loneliness and social connection (n=10)
  Improve social connections for those who are isolated
- Role in prevention (n=11)
  Potential role in prevention of mental health disorders through strengthening social connections
  Cheaper cost might mean rolled out earlier to help in prevention

**Threats**

- Patient protection from adverse unintended consequences (n=5)
  Those providing activities may not have the patients’ best interests
  No clear way that patients are being protected from outsiders
- Confidentiality and data protection (n=10)
  Data may be sold for profit
  Data may not be kept safe
  Hackers may access data
- Bias (n=10)
  Educated middle-class groups more likely to use technology to their advantage than those who need services
  Some groups may be favored over other either through the algorithm being inherently biased or access only being available in neighborhoods who can afford to invest in a digital solution
- Not helpful for some groups (n=5)
  Not helpful for all mental health conditions
  Many people are not online and do not wish to be, some of the groups who need social prescribing the most are among these

### Strengths

The expert group identified the main strength of DSPTs as being quick to start, whereas the nonexpert group perceived the main strength as their potential cost-effectiveness.

Both experts and nonexperts suggested that DSPTs would be faster to use; the nonexpert group suggested that using a digital platform would make the process of social prescription faster partly through a reduction in paperwork for those prescribing the activity. Both the expert and nonexpert groups commented on DSPTs being easy to use and having an improved user experience. Nonexpert participants suggested that reasons for these included users being able to clearly track their referral through the platforms, the platforms providing an *on-demand* service, and that all activity information would be consolidated in one place. They further suggested that younger people would find this way of accessing services easier to navigate than traditional methods. Both expert and nonexpert groups also suggested that DSPTs could be used to help individuals feel more connected to their local community.

The nonexpert group suggested that cost-effectiveness would be a significant advantage of DSPTs, whereas none of the expert participants commented on their cost-effectiveness. The nonexpert group suggested that although digital social prescription would use an algorithm for matching patients, there would be fewer people who would need to be involved in the social prescription process, which may result in the process being less costly.

### Weaknesses

Experts identified the main weakness of DSPTs as having a high dropout rate, whereas nonexpert groups were concerned that certain groups would find technology particularly difficult to use.

Both experts and nonexperts commented on the loss of interest and high dropout rates of patients using DSPTs. One expert suggested that patients may be fatigued with technology *solving* problems, and nonexperts additionally suggested that patients may not understand the point of DSPTs and may therefore not be motivated to continue using it. Both experts and nonexperts identified that DSPTs may be difficult for certain groups to use. These groups included older people, people with physical health disabilities, people from lower socioeconomic groups, and people with cultural or language barriers. Both experts and nonexperts also commented on the difficulty of maintaining the updated lists of local activities.

Experts commented on specific issues related to the technology used in facilitating digital social prescriptions. Experts commented on the difficulty in health care services being able to keep up with new developments in technology. They also commented on the potential interference on platforms by bots and trolls, which may affect the authenticity of participation.

Nonexperts raised concerns about DSPTs being inappropriate for those experiencing serious mental illness or where activities on offer may not be culturally appropriate. Several participants commented on digital social prescriptions resulting in a possible loss of human connection, perceived as inappropriate by patients and their families. A delay in appropriate treatment has also been cited as a potential weakness.

### Opportunities

Both experts and nonexperts felt that the main opportunity relating to digital social prescription was an increased access to mental health care. Experts particularly felt that this may be of particular benefit to *hard-to-reach* groups, including those from poorer socioeconomic backgrounds or those with other physical health comorbidities. Both experts and nonexperts perceived DSPTs as a potential help to prevent loneliness and improve social connection.

One expert commented on digital social prescription helping with resource efficiency by freeing up resources that could be directed to those experiencing significant mental illness. Nonexperts considered DSPTs to play a role in the prevention of mental health disorders.

### Threats

Both experts and nonexperts were concerned with data protection, confidentiality, and the potential monetization of data. Both experts and nonexperts also commented on the potential of bias resulting in a widening of health outcomes among different groups of individuals. This may be due to affluent middle-class individuals being the early adopters of new technology or due to the algorithms used in the DSPTs being inherently biased against certain groups. Nonexpert participants also commented that digital social prescriptions may be funded in certain areas, but this may not be the case in other areas.

Nonexperts considered that some individuals who would benefit from social prescription may not want to use new digital technologies to access activities. They also note that digital social prescriptions may not be beneficial for all mental health conditions. Some participants expressed concern regarding unintended consequences of digital social prescription; for example, if the activity involved patients volunteering at a coffee shop, then these patients may be exploited as free labor.

Experts additionally suggested that digital social prescriptions may be seen as a fad by clinicians and rejected.

## Discussion

### Principal Findings

From the consultation of the various participants, our study proposes a definition for digital social prescription: “Digital social prescription refers to social prescription that has been facilitated through the use of technology, such as mobile phone apps and online platforms intended to benefit users.”

The main perceived benefits of DSPTs were improved access to mental health care, fast adoption by users, and cost-effectiveness. Other perceived benefits included improved user experience, helping users feel more connected to their local communities, and potential prevention of loneliness and serious mental illness. There appeared to be significant crossover with regard to the perceived benefits of DSPTs from both experts and nonexperts. The main exception to this was cost-effectiveness, which was considered a significant benefit from nonexperts but was not commented on by the expert group.

The main challenge of DSPTs identified from our questionnaire was a poor engagement with such tools and certain groups finding the technology difficult to use. Other challenges include the DSPT being viewed as inappropriate by both patients and clinicians, certain groups being effectively excluded from using these tools, unintended negative consequences for patients, and concerns with confidentiality and data protection. Experts also commented on the difficulty faced by health care providers in keeping up with developments in technology and security, which may include issues relating to data hacking and interference from artificial intelligence–powered bots or trolls. The responses to the potential challenges from DSPT between experts and nonexperts were broadly similar; however, experts emphasized the challenges of technology more than nonexperts. Interestingly, almost all the expert respondents also commented on the high dropout rates of DSPTs, which may reflect their own experiences from working in the field and their concerns with user engagement.

### Strengths and Limitations

To our knowledge, this is the first qualitative study to explore the potential benefits and challenges of digital social prescription and suggests a definition of digital social prescribing that originated from such views. Our study compared the responses of a purposively selected sample of experts in digital mental health with those of nonexperts. The overall sample included a range of different ages, genders, nationalities, and occupations. By comparing the views of experts with nonexperts, we were able to identify key similarities and differences in their perspectives and views on digital social prescription, which, for the most part, were largely similar.

The main limitation of this study was that it had a small sample size. In addition, while focusing on incorporating views of experts and nonexperts (ie, the general public), there might have been other stakeholders, such as clinicians, patients, and caregivers, who we have not particularly targeted in this study. This would be an important area of further research, particularly as the use of digital tools in health care has become more prevalent. It is also worth noting that none of the expert cohort were older than 50 years, which may skew the views provided.

### Comparison With the Literature

The discussion of these findings was organized to reflect the themes that emerged in our study. The themes that were mentioned most frequently are discussed first.

It is important to note that as the majority of studies relating to social prescription refer to nonpharmacological prescription of exercise (exercise groups, gym programs, etc), most of the available literature concerns nonmental health–specific social prescription programs. Nevertheless, they provide a basis for understanding some of the core discussions regarding implementation and barriers to social prescription, which may also be relevant to digital social prescribing for mental health.

A key benefit of DSPTs identified by both experts and nonexperts was improved access to mental health care. Access to mental health care is a significant issue worldwide. The Five Year Forward View of Mental Health published in 2016 identified that approximately 15% of those with anxiety and depression were being seen by Improving Access to Psychological Therapy services [19]. The provision of services in low- and middle-income countries is even more sparse, with estimates suggesting that up to 90% of individuals living with mental health disorders are receiving no mental health care [20]. Access to smartphones has been a global phenomenon, and there has been a considerable interest in delivering mental health care through mobile phone technology [21]. Young people have been shown to adopt new technologies quickly and to use mobile phone technology in the event of sickness, personal health crises, or in response to health concerns of others [22]. In the United Kingdom, a majority of mental health conditions are managed through primary care, and it has been suggested that the use of technology may allow for more options of self-referral with automated or semiautomated interventions, thereby improving access [23].

Cost-effectiveness was perceived as one of the main benefits of digital social prescriptions by nonexpert participants in this study. Cost-effectiveness and social prescriptions have been a hotly debated topic over the past decade. Some studies have indicated that social prescription may result in fewer hospital and GP appointments, thereby translating into reduced costs for the National Health Service [7]. However, critics have suggested that there is a poor evidence for sustained improved health care outcomes [24,25] and that social prescription programs that have demonstrated positive health outcomes incur a higher cost than traditional care [26]. A systematic review of physical activity interventions in primary care showed that interventions ranged from £304 (US $425) to £75,982 (US $106,346) per quality-adjusted life year depending on the scheme intensity [27]. Digital social prescription may provide a greater efficiency in some respects to matching individuals with activities, but if the bulk of the cost depends on how individual programs are run, then the use of a digital platform may only have a marginal effect on costs for social prescription programs.

One of the main barriers in assessing social prescription programs is that the programs delivered by third-sector organizations often have limited funding, and it is therefore difficult to gather data on outcomes over a sustained period [28]. It is likely that this same problem will exist with digital social prescribing programs, as the activities that are matched with patients would also be largely provided through third-sector organizations.

Interestingly, the study participants did not comment on the intrinsic benefits and functionality that technology may have beyond being quick and easy to use. A review conducted by Husk et al [29] did not identify speed and efficiency as important factors leading to the successful use of social prescription programs, and human factors such as support from their link worker or practical support, such as free travel for activities, mattered much more to participants. There may, however, be opportunities provided by using digital means to access social prescriptions. Hollis et al [30] described the potential of mobile phone apps having embedded validated measures such as the Patient Health Questionnaire-9 depression scale as well as the option for patients to track their symptoms over time. With respect to DSPTs, this may also mean that large amounts of user data that can be used to evaluate the effectiveness of these tools can be collected quickly and accurately.

Adherence to DSPTs was identified as the main challenge by both experts and nonexperts. Indeed, adherence to traditional social prescription programs has also been shown to be challenging. Pavey et al [31] conducted a systematic review of the uptake and adherence to exercise referral schemes, which are the most common social prescription in the United Kingdom. They identified that the pooled level of adherence to exercise referral schemes was only 49% in observational studies and 43% in randomized controlled trials. In studies examining factors that improve adherence to social prescription programs, the relationship between navigators and patients has been shown to be one of the most important factors facilitating social prescription [32,33]. The skill of those conducting the activity also appears to be an important factor for adherence [34,35] as well as patients being able to see positive results from undertaking activities [36]. Given the existing literature, one can assume that a purely digital social prescription platform, in which there is no direct human contact, may result in even poorer adherence. However, a digital platform may allow participants to record key data, such as sleep and mood, and improvements in these parameters may improve adherence.

Several barriers to using digital social prescriptions were also described. Cultural and religious factors are likely to play an important role in determining whether a social prescription will be effective. In several cultures, seeking help for mental health conditions can often be stigmatizing [37], and some activities such as mixed-sex swimming may be seen as inappropriate in the context of an individual’s culture. Language may also be a significant barrier in allowing individuals to participate in a prescribed activity if the DSPT is only available in English. In addition, digital barriers were also described by participants. Older people in the United Kingdom have been shown to experience high rates of loneliness as compared with other groups in the society [38]; however, official Office for National Statistics data in 2019 showed that from those aged ≥75 years who participated in the survey, less than half used the internet [39]. Ethnicity has also been shown to contribute to the digital divide, with studies showing that Black, minority, and ethnic backgrounds are more likely to access computers outside their own homes as compared with White individuals [40]. This brings with it the challenge of ensuring adequate privacy in engaging with internet-based content related to an individual’s mental health.

Data protection and information sharing are important factors to consider in digital social prescriptions. It also appears to be a concern for consumers. In a 2017 survey, confidence in the data security of technology companies declined from 31% in 2016 to 24% in 2017 [41]. Confidentiality is an important tenet of medicine; however, in practice, there are many scenarios in which information sharing between parties is necessary to provide the best care for a patient. Guidelines relating to social prescription have indicated that it is the responsibility of the referrer to transfer any relevant information to the person conducting the nonmedical activity [42]. Despite this, survey data [41] have indicated that patients are much more averse to sharing their data with nonphysicians, even if these parties are integral to the delivery of patient care. Clear guidelines explaining how data are used and stored would be required to ensure that the consent from patients is valid. It would also be necessary to consider how these security rules would be enforced and what remedies should be offered to those affected by security breaches.

Algorithmic programming is central to the apps that we use today and is likely to be used in the development of a DSPT. These algorithms might result in potential race discrimination, gender discrimination, and ageism [43,44]. This may also be an important consideration with regard to a DSPT. Existing psychiatric risk assessment tools that have been shown to have poor accuracy [45] may be integrated into digital social prescribing software, further resulting in an effective discrimination against certain groups. Furthermore, clinicians who may be involved in designing these tools may introduce their own biases, which could include greater patient restrictions, particularly for those of certain ethnic backgrounds [46]. Organizations, including the Open Data Institute, are considering the potential ethical implications arising from the use of digital tools and have suggested the use of ethical frameworks such as the Data Ethics Canvas [47] to address these issues.

Although there have been no known studies directly looking at the unintended consequences of digital social prescription, bridging the online and offline worlds can create risks, and in cases where things might go wrong, liability may be an issue for both clinicians and software developers. There has been some discussion of the potential negative consequences relating to social prescription [48], which includes patients becoming stressed by the commitment required or becoming so consumed in an activity that they neglect other key aspects of their life and well-being.

### Implications for Practice, Research, and Policies

DSPTs may be a helpful method for delivering nonmedical activities to those with mental illness. There are various types of DSPTs with their differences, although with a commonality of providing patients with nonmedical activities that are available in a patient’s local area. The use of such DSPTs may result in greater accessibility of activities for patients and may be more cost-effective than traditional social prescription methods.

There are several challenges associated with digital social prescriptions. First, digital social prescriptions may not be appropriate for all patients. A careful consideration of symptomatology and patient expectations must be considered before making any universal recommendations. Barriers to using digital social prescriptions are likely to exist. This may include cultural and language barriers, difficulty with using the technology due to unfamiliarity, or difficulty with using the platform due to physical impairment. Cost may also be a prohibitive factor. These barriers need to be studied in more detail, and steps should be taken to improve access to digital social prescriptions. Issues relating to patient confidentiality and data protection are likely to arise in the development of DSPTs. These issues should be considered at every stage of the development and implementation of digital social prescription programs.

Although digital social prescriptions may be of benefit to patients, there is not enough evidence to substantiate this. Research looking at short-term and long-term outcome measures, such as clinical impact and cost-effectiveness, is required to identify the true benefit. Given that adherence to DSPTs was identified as the main perceived challenge, research into how adherence may be improved would also be important. On the basis of the data collected from this research, decisions can be made as to whether DSPTs should be used more widely in mental health care.

### Conclusions

Digital social prescriptions may be able to provide important opportunities and help to reduce the burden of distress in patients. Important patient considerations ranging from the appropriateness of an activity to patient discrimination will need to be carefully considered in the design and implementation of this technology. More evidence is needed to further support the advancement of digital social prescribing, but with more rigorous research and respect for data ethics, this may be a significant advancement in 21st century medicine.

## Appendix

Multimedia Appendix 1 Digital social prescribing questionnaire used in the study.

## Abbreviations

DSPT: digital social prescription tool
GP: general practitioner
SWOT: strengths, weaknesses, opportunities, and threats
