# Correction: Opportunities and Challenges for Digital Social Prescribing in Mental Health: Questionnaire Study

**DOI:** 10.2196/29041

**Published:** 2021-03-29

**Authors:** Shivani Patel, Gerry Craigen, Mariana Pinto da Costa, Becky Inkster

**Affiliations:** 1 South London and Maudsley NHS Trust London United Kingdom; 2 Department of Psychiatry, Faculty of Medicine University of Toronto Toronto, ON Canada; 3 Centre for Mental Health University Health Network – Toronto General Toronto, ON Canada; 4 Institute of Psychiatry, Psychology and Neuroscience King’s College London London United Kingdom; 5 Institute of Biomedical Sciences Abel Salazar University of Porto Porto Portugal; 6 Department of Psychiatry University of Cambridge Cambridge United Kingdom; 7 Finance and Economics Programme The Alan Turing Institute London United Kingdom

In “Opportunities and Challenges for Digital Social Prescribing in Mental Health: Questionnaire Study” (J Med Internet Res 2021;23(3):e17438) the authors noted three errors.

In the originally published article, affiliation 1 was not applied to author Mariana Pinto da Costa, and the affiliation *“Centre for Mental Health, University Health Network – Toronto General, Toronto, ON, Canada”* was not added for author Gerry Craigen.

The author's affiliation section was originally published as follows:

Shivani Patel^1^, MRCPsych; Gerry Craigen^2^, MD; Mariana Pinto da Costa^3,4^, MD; Becky Inkster^5,6^, DPhil

^1^South London and Maudsley NHS Trust, London, United Kingdom

^2^Department of Psychiatry, Faculty of Medicine, University of Toronto, Toronto, ON, Canada

^3^Institute of Psychiatry, Psychology and Neuroscience, King’s College London, London, United Kingdom

^4^Institute of Biomedical Sciences Abel Salazar, University of Porto, Porto, Portugal

^5^Department of Psychiatry, University of Cambridge, Cambridge, United Kingdom

^6^Finance and Economics Programme, The Alan Turing Institute, London, United Kingdom

The corrected author's affiliation section appears as follows:

Shivani Patel^1^, MRCPsych; Gerry Craigen^2,3^, MD; Mariana Pinto da Costa^1,4,5^, MD; Becky Inkster^6,7^, DPhil

^1^South London and Maudsley NHS Trust, London, United Kingdom

^2^Department of Psychiatry, Faculty of Medicine, University of Toronto, Toronto, ON, Canada

^3^Centre for Mental Health, University Health Network – Toronto General, Toronto, ON, Canada

^4^Institute of Psychiatry, Psychology and Neuroscience, King’s College London, London, United Kingdom

^5^Institute of Biomedical Sciences Abel Salazar, University of Porto, Porto, Portugal

^6^Department of Psychiatry, University of Cambridge, Cambridge, United Kingdom

^7^Finance and Economics Programme, The Alan Turing Institute, London, United Kingdom

In the “Data Analysis” section, a correction has been made to clarify the author order referenced in the text.

The following sentence appeared in the originally published manuscript:

The first author (SP) coded all the material, and the second author (MP) reviewed all the data to ensure the consistency and credibility of the coding and grouping [18].

This sentence has been corrected to:

The first author (SP) coded all the material, and the third author (MP) reviewed all the data to ensure the consistency and credibility of the coding and grouping [18].

The correction will appear in the online version of the paper on the JMIR Publications website on March 29, 2021, together with the publication of this correction notice. Because this was made after submission to PubMed, PubMed Central, and other full-text repositories, the corrected article has also been resubmitted to those repositories.

