# Supplementary material for: Opportunities and Challenges for Digital Social Prescribing in Mental Health: Questionnaire Study
Source: J Med Internet Res. 2021 Mar 9;23(3):e17438. doi: 10.2196/17438 (PMC7988390; doi:10.2196/17438)
Supplement: Multimedia Appendix 1 [file jmir_v23i3e17438_app1.docx]

**Multimedia Appendix 1.** Digital social prescribing questionnaire used in the study.

| **DIGITAL SOCIAL PRESCRIPTION QUESTIONNAIRE**  The following questions are about digital social prescription in mental health. In order to better understand your views on the role that digital social prescription may play in mental health, we have prepared a short questionnaire, aiming to do a SWOT analysis on digital social prescribing, looking at its strengths, weaknesses, opportunities and threats. This should take no longer than 5-10 minutes to complete. There are no right or wrong answers - in all these questions we are only asking for your personal view.  Below is a brief outline as to how a digital social prescription tool may be used:  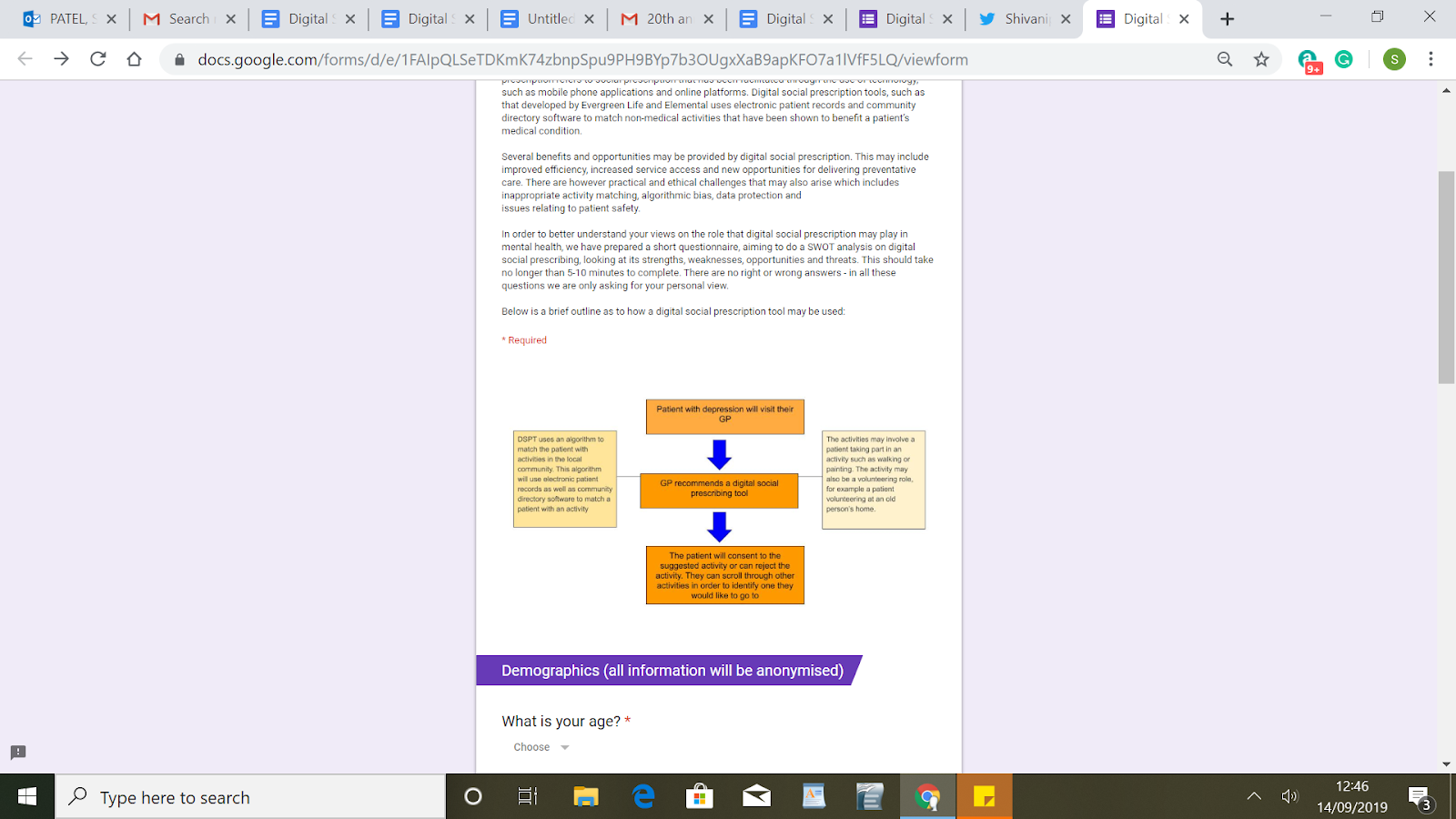  **Demographics (all information will be anonymised)**   1. What is your age? 2. What is your gender? 3. What is your nationality? 4. What is your current occupation/job?   **Questions on Digital Social Prescription**   1. How would you define digital social prescription? 2. What do you think could be the benefits of using a specific digital social prescription tool? 3. What do you think could be the difficulties that may arise from using a specific digital social prescription tool? 4. What opportunities do you think that digital social prescription, in general, may be able to provide? 5. What do you think are the potential challenges arising from digital social prescription? |
| --- |
